# Chloride fertilization increased maize shoot chloride levels and yield depending on soil moisture conditions

**DOI:** 10.1038/s41598-026-68369-x

**Published:** 2026-08-25

**Authors:** Mirjam Koch, Christoph-Martin Geilfus, Muhammad Waqas, Sonoko D. Bellingrath-Kimura, Moritz Reckling

**Affiliations:** 1 Department of Pomology, Hochschule Geisenheim University, Von-Lade-Str. 1, 65366 Geisenheim, Germany; 2 Department of Soil Science and Plant Nutrition, Hochschule Geisenheim University, 65366 Geisenheim, Germany; 3 State Key Laboratory of Desert and Oasis Ecology, Xinjiang Institute of Ecology and Geography, Chinese Academy of Sciences, Urumqi, 830011 China; 4 Leibniz Centre for Agricultural Landscape Research e.V, Eberswalder Straße 84, 15374 Müncheberg, Germany; 5 Faculty of Life Science, Thaer-Institute of Agricultural and Horticultural Sciences, Humboldt-University of Berlin, Unter den Linden 6, 10099 Berlin, Germany

**Keywords:** Chloride, Silage maize, Drought tolerance, Osmotic adjustment, Field trial, Ecology, Ecology, Environmental sciences, Plant sciences

## Abstract

Growing crops on sandy soils becomes especially difficult during summer when rainfall is scarce, making drought conditions even more severe. The micronutrient chloride (Cl^−^) can accumulate in plant shoot tissue reaching contents that are typical for macronutrients, assumed it is supplied and taken up in appropriate amounts. When this happens, it can act as an osmoticum, facilitating shoot hydration, reported previously under drought. So far, this cascade of events was only reported under in-door conditions with pot experiments and to the best of our knowledge, not under field conditions. Under field conditions, uncontrollable environmental effects may limit the positive processes. Therefore, we conducted a field trial to investigate, how chloride application may enhance drought tolerance during the whole growth period of maize grown for silage. The field trial was conducted on a sandy soil in northeastern Germany subjecting maize to different Cl^−^-fertilization treatments (0, 125, and 250 kg Cl^−^ ha^− 1^ a^− 1^; given together with calcium as counter cation) in the years 2020 and 2021. The setup allowed for the assessment of Cl^−^-fertilization of (i) water status, via shoot water content and rain use efficiency (RUE; kg FM ha^− 1^ mm^− 1^ per vegetation period), and (ii) biomass yield under field conditions. We found a significant increase of Cl^−^-content in shoot tissue following the Cl^−^-fertilization (125 and 250 kg Cl^−^ ha^− 1^, respectively) in both years. However, only in the year 2021, the higher Cl^−^ plant tissue content (4.6 g Cl^−^ kg^− 1^ DM under a treatment of 250 kg Cl^−^ ha^− 1^) was associated with significantly higher shoot water content at harvest and rain use efficiency. In the year 2021, were precipitation in spring was sufficient for the crop water demand, chloride fertilization supported shoot hydration by acting as an osmoticum.” In this year plants treated with 250 kg Cl^−^ ha^− 1^ showed a significantly higher fresh biomass yield. However, the improved water status did not result in a stimulated carbon assimilation. But chloride-fertilization decreased the dry matter content of silage maize. For the first time, this study showed that Cl^―^-fertilization supported silage maize to cope with water scarcity on sandy soils.

**Supplementary Information:**

The online version contains supplementary material available at 10.1038/s41598-026-68369-x.

## Introduction

At the beginning of the twenty-first century, 2003 was classified as the driest year in Europe since the severe drought of 1540^1,2^. The drought in 2003 mainly affected France, northern Italy, western Switzerland and Germany^1^. After 2003 the year 2018 was invoked as the driest year since ~ 500 years, causing problems in Austria, Switzerland and Germany^3^. Only four years later, 2022 is known as the driest year since 1540^4^. Compared to 2018, the drought in 2022 affected almost the whole European continent while the drought in 2018 affected especially Central European countries. The 2022 event was expected to surpass 2018 in heat and dryness, causing major socio-economic impacts, including water restrictions in Italy and France and severe effects on ecosystems and agriculture^4^. To summarize, it can be stated that since the beginning of the 2000s summer drought events are more frequently reported for Central and Southern Europe compared to the 20th century^3,4^.

After wheat, silage maize is the most important crop in Germany, at least in terms of area^5^, being especially used as feed for livestock and as energy crop for biogas production. Like many other crops, silage maize that experiences drought shows high yield losses. In dependence on the intensity and the duration of water shortage and the phenological stage, yield loss may vary between 20 and 50%; the phenological stages flowering and grain filling (approximately BBCH 60–80) are reported to be the most sensitive towards water deficits^6^. Since spring and early summer drought are expected to increase in central Europe^7^, this is a high risk for maize production in Germany.

In 2018, the lowest silage maize yields in Germany were recorded in Brandenburg with 21.4 t/ha, while Bavaria reported the highest yields at 46.2 t/ha, i.e. more than twice as much^8^. This huge contrast reflects Brandenburgs generally lower annual precipitation and higher temperatures compared to other federal states such as Bavaria or North Rhine-Westphalia^9^. Brandenburg has sandy soils revealing a low water holding capacity and being even more affected by water shortages.

Agronomic strategies that improve water use efficiency are needed, especially in regions prone to drought like Brandenburg. These strategies should help crops cope more effectively with limited water availability and increasing drought stress. A largely unexplored approach that has not been tested under field conditions could be the targeted fertilization of crops with chloride (Cl^−^). Although Cl^−^ is an essential micronutrient, it can accumulate in leaves typically seen with macronutrients and unfold benefical functions. Many studies found that Cl^−^ is acting as an osmoticum at such leaf tissue content: The accumulation of Cl^−^ in leaves results in a decreased osmotic potential leading in turn to an increased water content in leaves^10,11^. In consequence, in tobacco (*Nicotiana tabacum* L. var. Habana) plants a turgor-driven leaf cell-expansion was observed resulting in increased leaf biomasses^10,12^. Similarly, Koch et al.^13^ determined a higher shoot water content following a Cl^−^-accumulation in leaves of potato (*Solanum tuberosum* L.) plants, however without increasing nor decreasing effects on tuber yield. Cui et al.^14^ have shown that the salt tolerance of the xerophytic plant *Pugionium cornutum*—belonging to the family of Brassicaceae—is based on the osmotic properties of Cl^−^. The authors could demonstrate that a suppression of Cl^−^-accumulation in the shoot resulted in reduced plant growth, reduction of the shoot water content and a decreased photosynthetic rate. These desribed findings suggest that Cl^―^ can act as an effective osmoticum, improving drought tolerance if stored in plant tissues before stress occurs. To avoid reduced water uptake or salt damage, Cl^−^-levels must be carefully managed through controlled fertilization. Chloride can be introduced in agricultural field as fertilizer, accompanying potassium (KCl) or ammonium (NH_4_Cl) or calcium (CaCl_2_). Compared to other anions Cl^−^ differs fundamentally from other anions such as nitrate ($\:{NO}_{3}^{-}$), sulfate ($\:{SO}_{4}^{2-\:}$) or phosphate ($\:{PO}_{4}^{3-}$). Chloride largely remains in its inorganic form and does not consume any metabolic costs such as, for instance, NO_3_^−^, which first needs to be reduced and assimilated for plant uptake and usage. Besides, Cl^−^ has a higher mobility compared to other multivalent anions such as$\:{SO}_{4}^{2-\:}$ and$\:{PO}_{4}^{3-}$ and is easily transported to the shoots where it can act directly as osmoticum while other anions are serving more metabolic or structural roles^15^.

Overall, Cl^−^ appears to be an effective, low-cost osmoticum with the potential to enhance plant performance under water deficit. However, to the best of our knowledge, positive effects of Cl^−^ on plant growth have thus far been demonstrated mainly under controlled conditions, such as pot experiments or hydroponic systems. In contrast, field conditions are characterized by strong spatial and temporal variability in soil water availability, temperature, and nutrient supply, as well as by complex interactions between these factors. Under such conditions, crop responses to Cl^−^ fertilization may be modified or masked by drought intensity, soil texture, rooting depth, and interactions with other nutrients. As proposed agronomic measure, it is necessary to analyse the robustness of the effects of Cl^−^ under variable environmental conditions. Thus, the present study aimed to examine whether a Cl^−^-fertilization is able to increase the yield of silage maize grown on a sandy soil in Brandenburg under rainfed field conditions. We hypothesize that Cl^−^-fertilization will result in (i) increased maize water uptake and (ii) in increased fresh and dry matter yields.

## Material und methods

### Experimental site and design

**Fig. 1:**
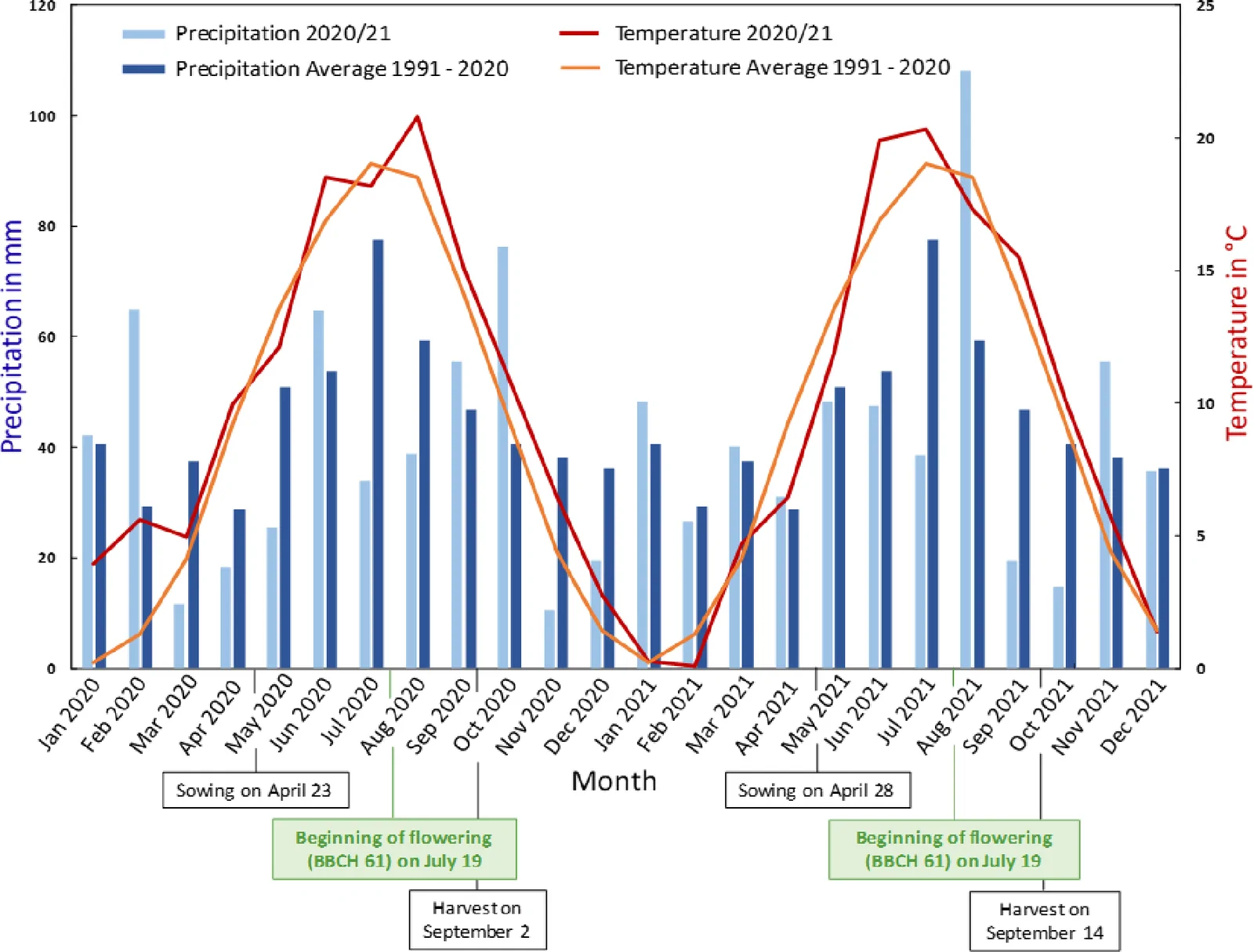
Monthly precipitation and temperature patterns in 2020 and 2021 relative to the 1991–2020 climate normal. Light blue bars indicate monthly precipitation (mm) in 2020 and 2021, dark blue bars represent the long-term monthly average precipitation for 1991–2020. Red lines show monthly mean temperature (°C) in 2020 and 2021, and orange lines indicate the corresponding long-term average temperature for 1991–2020. Important crop management dates and key phenological stages are indicated for both years.

Field experiments were established at the Leibniz Centre for Agricultural Landscape Research (ZALF) in 2020 and 2021, in NE Germany (field in 2020: 52º 30′ 59″ N, 14º 07′ 37″ E, and field 2021: 52º 31′3″ N, 14º 07′51″ E, 63 masl). The soil particle size distribution was 80% sand, 15% silt and 5% clay in the 0–60 cm layer. The soil had a field capacity volumetric water content of approximately 15% and a permanent wilting point content of 4% in the 0–60 cm layer. The soil chemical properties and plant-available nutrients are given in Table 1. For soil chemical analysis soil samples were taken in 0–30 cm depth on 10th and 15th of November in 2020 and 2021, respectively. Per year all 36 plots were sampled with 5 cores per plot using a tractor mounted soil auger machine. Soil samples were air dried, sieved and a representative portion was analyzed unsing Kjeldahl digestion method and flame photometry by ZALF central lab.The experimental area has a score of 25–32 points within the German soil quality rating, which has a range from < 20 (marginal and difficult to crop) up to 100 (most fertile). The region has a central European climate with an annual mean air temperature of 9.4 °C, precipitation of 567 mm and potential evapotranspiration of 716 mm. On average, winter, spring, summer and autumn months receive 110, 144, 166 and 121 mm, respectively^16^. Figure 1 shows the monthly average precipitation and temperature of the experimental years 2020 and 2021 as well as the average precipitation and temperature of the period 1991 to 2020 together with important crop management dates and key phenological stages in 2020 and 2021.

**Table 1:** Soil chemical properties and plant-available nutrient concentrations determined by CAL (calcium acetate lactate) extraction for phosphorus and potassium, by CaCl_2_ extraction for magnesium, by ALE (ammonium lactate extraction) extraction for calcium, and soil pH for the years 2020 and 2021. Total carbon and nitrogen are given in %. Phosphorus, potassium, magnesium and calcium are given in mg 100 g^− 1^ soil. Chloride is given in mg kg^− 1^ soil.

Year	Total carbon	Total nitrogen	Phosphorus	Potassium	Magnesium	Calcium	Chloride	pH
2020	0.82	0.07	7.3	10.8	3.4	67.4	0.91	7.1
2021	0.52	0.05	5.1	4.5	3.8	59.4	0.43	6.5

The experiment was a randomized complete block design with six replications. The unit plot size was 48 m^2^ (6 m by 8 m). The factor was calcium chloride (CaCl_2_) fertilzation by applying it to the soil before sowing in March with three levels: 0 kg ha^− 1^, 125 kg ha^− 1^ and 250 kg ha^− 1^. Chloride-fertilization was the experimental factor and was applied at 0, 125, and 250 kg chloride ha^−^¹ to represent a non-fertilized control, an intermediate chloride supply, and a high chloride supply intended to promote shoot Cl^−^ accumulation above the micronutrient requirement range without deliberately inducing salt stress. Thus, the selected rates were designed to test the physiological and agronomic response to Cl^−^ under field conditions rather than to define an optimized fertilization recommendation. The field received lime of the entire area with CaCO_3_ (product “Granukal” with 68% CaCO_3_) to reduce a potential Ca effect from the CaCl_2_ fertilizer and was tilled in March. CaCl_2_ fertilizer was incorporated together with the seed bed preparation before silage maize (*Zea mays* L.) sowing in April.

Silage maize var. KWS Robertino (FAO 230 maturity for silage) was sown with 9.5 germinating seeds per m^2^ and a 75 cm row spacing on April 23 and 28 in 2020 and 2021, respectively. Chemical weed control was performed post-emergence at the end of May and no further plant protection was applied. Maize received a mineral fertilizer after sowing (before emergence), with 140 kg nitrogen ha^− 1^ and 34 kg sulphur ha^− 1^ (product “Piasan” 25/6). No additional phosphorus and potassium fertilization was applied. Maize was harvested on September 2 and 14, in 2020 and 2021, respectively.

### Sample processing and lab analysis

After harvest, subsamples of shoot material were taken and there fresh weight were examined. Plant material was dried at 60 °C in a drying chamber to a constant weight and dry mass content % was calculated. A subsample of 50 g was milled and used in the central lab of ZALF for the nutrient analysis. To determine the plant nutrients (potassium, nitrogen, phosphorus, magnesium, calcium and sodium) subsamples from each plot harvested were first milled to a sieve size of 1 mm, from which a representative portion was analyzed unsing Kjeldahl digestion method and flame photometry (AAS-iCE 3300, Gallery Plus, ThermoFisher Scientific GmbH Microgenics GmbH, Hennigsdorf). Chloride determination in the plant tissue was performed using a titration method with a chloride-selective electrode. To accurately determine chloride levels, a calibration sample was used and verifyed the accuracy and reliability of the analytical method established in the central lab of ZALF.

### Rain use efficiency

The rain use efficiency was calculated by dividing the fresh mass yield (dt ha^− 1^) by the precipitation over the entire growth period in 2020 (180.5 mm) and in 2021 (256 mm), respectively, starting with sowing in April and ending with harvest in September (Fig. 1).

### Statistics

All analyses were performed in R version 4.5.0, 2025, utilizing the packages *readxl*, *nlme*, and *emmeans*. Model fit quality was additionally evaluated using the *piecewiseSEM* package. Linear mixed-effects models were fitted using the *nlme* package to investigate the effects of fertilization treatment and year, including their interaction. Relevant variables such as *YEAR*, and *FERTILIZING_CaCl2* were converted to factor variables for subsequent statistical modeling. Analysis of variance (ANOVA) was performed using type marginal tests to evaluate fixed effects significance. Pairwise comparisons between fertilization treatments, averaged over years, were conducted using Tukey-adjusted contrasts implemented via the *emmeans* package. Additionally, year effects within each fertilization level were assessed, as well as fertilization effects within each year, allowing for detailed interpretation of interactions when significant. A separate two-way ANOVA was performed followed by Tukey’s post-hoc tests to assess year-by-fertilization effects. Statistical significance was defined as *p* < 0.05. Results with 0.05 ≤ *p* < 0.10 were interpreted as marginal trends and are indicated by a superscript θ in figures and tables. Pairwise comparisons were based on Tukey-adjusted contrasts.

## Results

### Shoot chloride concent and shoot water content

**Fig. 2:**
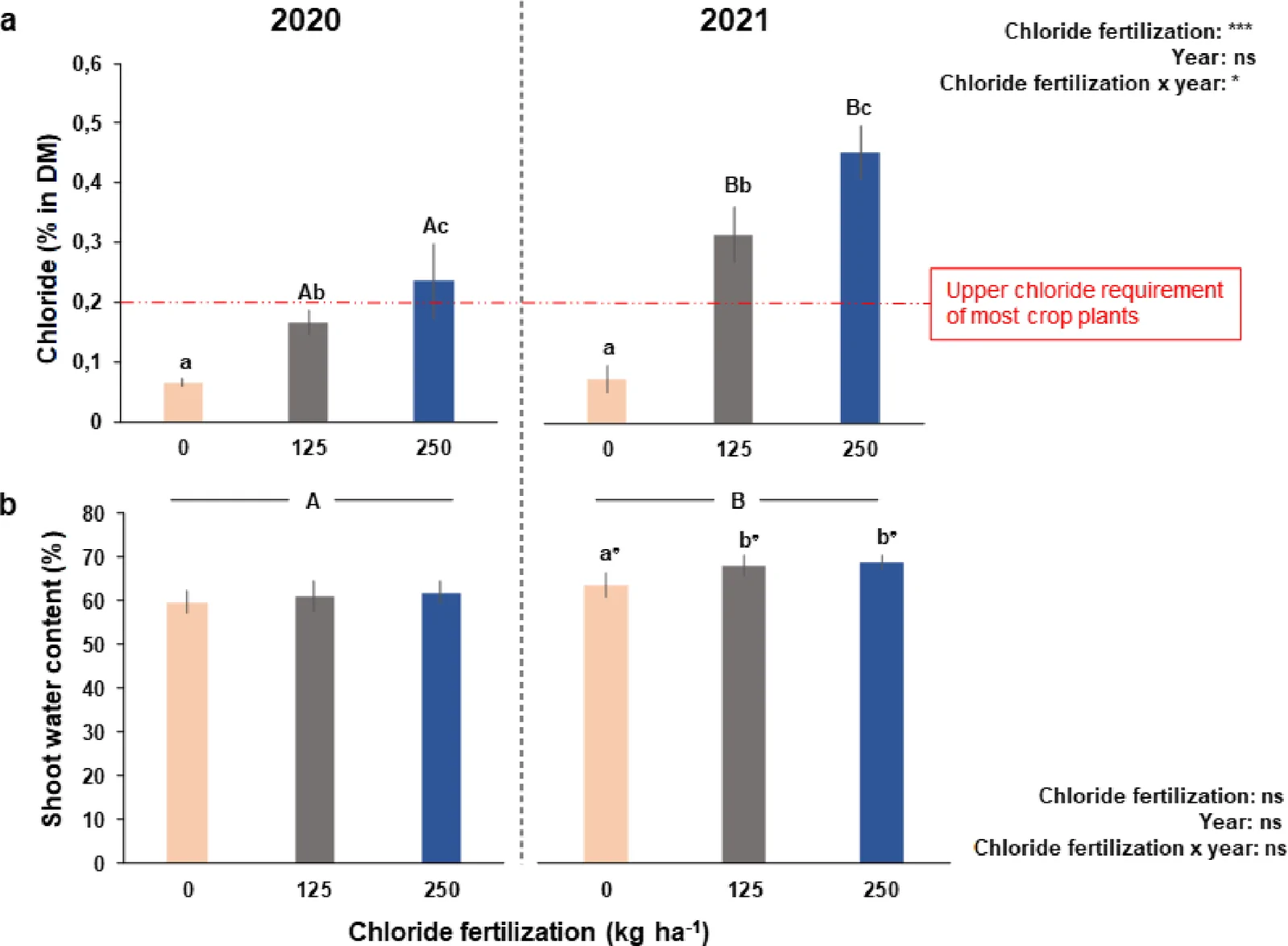
Chloride concentrations and shoot water content of silage maize in 2020 and 2021 under different chloride fertilization treatments. Chloride in % in dry matter (DM) in silage maize in the years 2020 and 2021 (**a**) and shoot water content in % at harvest in the years 2020 and 2021 (**b**) under three different chloride fertilizations (0, 125, and 250 kg ha^− 1^). *n* = 6, mean ± SE values. Red dotted line in (**a**) shows the upper requirement of chloride as a micronutrient of most crop plants. Results of the variance analyses with the fixed effects ʽchloride fertilizationʼ, ʽyearʼ, and their interaction are shown; significance levels are indicated by asterisks as follows:: *p* ≤ 0.05: *p ≤ 0.01: **p ≤ 0.001: ***; ns denotes not significant. Lowercase letters (**a**–**c**) indicate Tukey-adjusted pairwise comparison groups among chloride fertilization treatments within each year; means that do not share a lowercase letter differ at *p* < 0.05. Uppercase letters (**A**,**B**) indicate pairwise comparisons between years. A superscript θ indicates a marginal pairwise difference at 0.05 ≤ *p* < 0.10, whereas letters without θ indicate *p* < 0.05. Means sharing a letter are not significantly different at the respective significance level. The absence of a letter indicates that no significant or marginal pairwise difference was detected.

Cl^−^ fertilization resulted in significant increase in Cl^−^ content in silage maize biomass in both years (Fig. 2a). Chloride content doubled in response to 125 kg Cl^−^ ha^− 1^ compared to control plants (no Cl^−^ given) in the year 2020 and increased threefold in the year 2021. Both years showed an additional one-third increase in chloride accumulation when fertilized with 250 kg Cl^−^ ha^− 1^ compared to 125 kg Cl^−^ ha^− 1^.

In 2021, at harvest shoot water content was significantly increased in response to Cl^−^ fertilization with 125 and 250 kg ha^− 1^ compared to control plants, although significance was only reached at *p* < 0.1 (Fig. 2b). Additionally, in 2021 silage maize showed a significantly higher water content across all fertilization treatments compared to 2020 (Fig. 2b).

### Yield and rain use efficiency

**Fig. 3:**
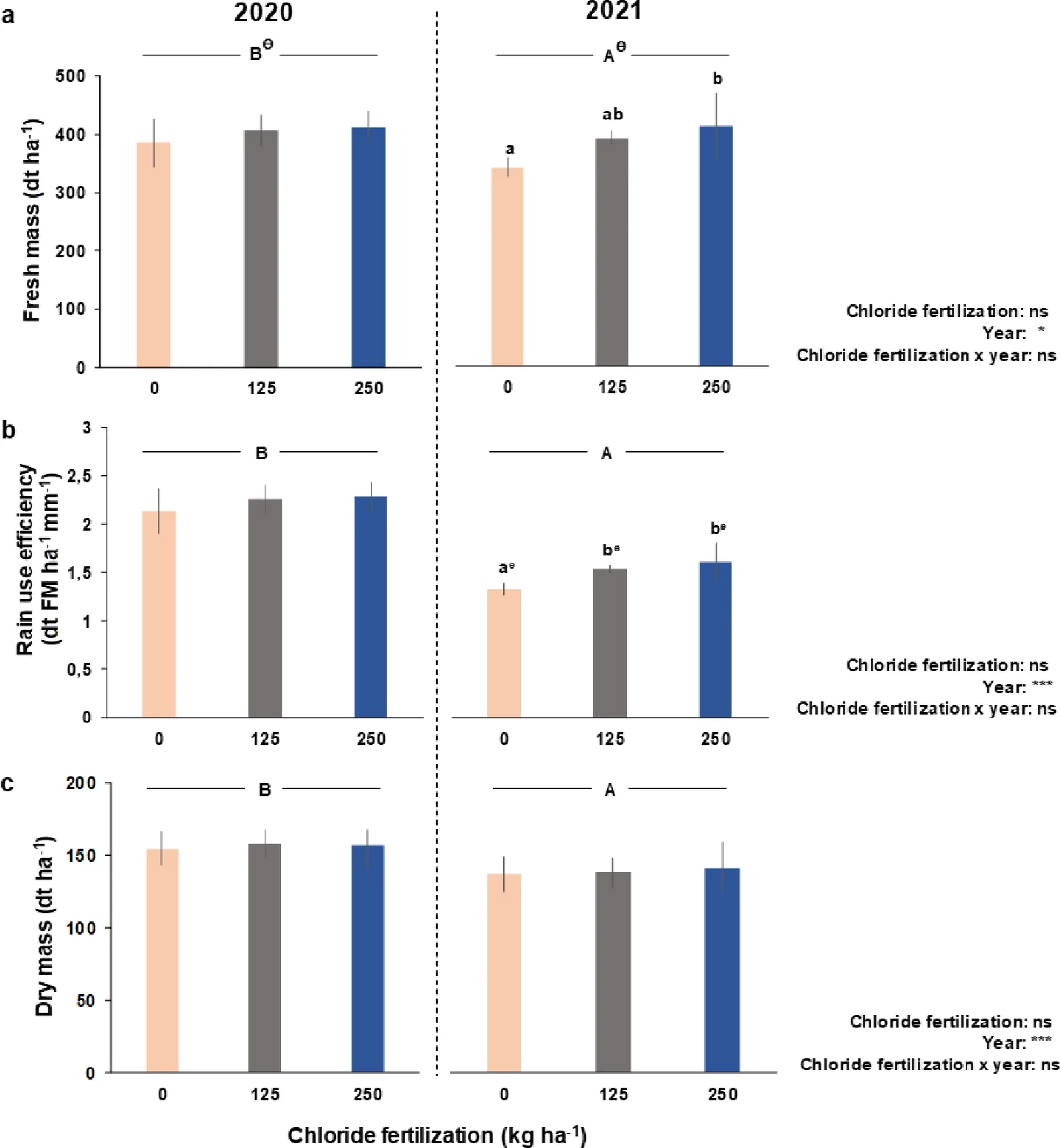
Fresh and dry mass yields and rain use efficiency of silage maize in 2020 and 2021 under different chloride fertilization treatments. Panels show fresh mass harvest yield in dt ha^− 1^ in the years 2020 and 2021 (**a**), rain use efficiency determined as fresh mass harvest yield in dt ha^− 1^ per precipitation in mm of the entire vegetation period (2020 or 2021) (**b**), and dry mass harvest yield in dt ha^− 1^ in the years 2020 and 2021 (**c**) under three different chloride fertilizations (0, 125, and 250 kg ha^− 1^). *n* = 6, mean ± SE values. Results of the variance analyses with the fixed effects ʽchloride fertilizationʼ, ʽyearʼ and their interactions; significance levels are indicated by asterisks as follows: *p* ≤ 0.05: *, *p* ≤ 0.01: **, *p* ≤ 0.001: ***; ns denotes not significant. Lowercase letters (a, b, c, where applicable) indicate Tukey-adjusted pairwise comparison groups among chloride fertilization treatments within each year; means that do not share a lowercase letter differ at *p* < 0.05. Uppercase letters (A, B) indicate pairwise comparisons between years. A superscript θ attached to either lowercase or uppercase letters indicates a marginal pairwise difference at 0.05 ≤ *p* < 0.10, whereas letters without θ indicate *p* < 0.05. Means sharing a letter are not significantly different at the respective significance level. The absence of a letter indicates that no significant or marginal pairwise difference was detected.

In 2021, biomass yield (fresh mass) was significantly higher in plants fertilized with 250 kg chloride ha^− 1^ compared to control plants (0 kg chloride ha^− 1^) (Fig. 3a). The year 2020 had overall higher fresh mass yields compared to the year 2021 (Fig. 3a) but fertilization did not reveal a significant effect.

The rain use efficiency was significantly higher (p-value < 0.1) when 125 and 250 kg Cl^―^ ha^− 1^ were fertilized, respectively, compared to control plants. The year 2020 demonstrated a significant higher rain use efficiency averaged over all fertilization treatments compared to year 2021 (Fig. 3b).

In both years, dry mass yield was not affected by the fertilization treatment. Similar to fresh mass yield, overall dry mass yield in 2020 was significantly higher compared to 2021 (Fig. 3c).

### Mineral nutrient content in silage maize

Cation contents increased with increasing Cl^−^-fertilization. Potassium (K), calcium (Ca) and sodium (Na) content were significantly higher under a Cl^−^-fertilization of 125 kg Cl^−^ ha^− 1^ compared to control plants in the year 2020. In the year 2021, magnesium (Mg) and Ca content were significantly increased under a fertilization treatment of 250 kg Cl^−^ ha^− 1^ compared to control plants. Thus, Cl^−^-fertilization consistently increased cation content in silage maize, with K, Ca, and Na responding in 2020, and Mg and Ca in 2021, indicating a year-dependent cation response pattern. Moreover, there were significant year effects: in 2020 the K, N, Ca and Na content were significantly higher compared to the year 2021 while the phosphorus (P) and Mg content were significantly lower.

**Table 2:**
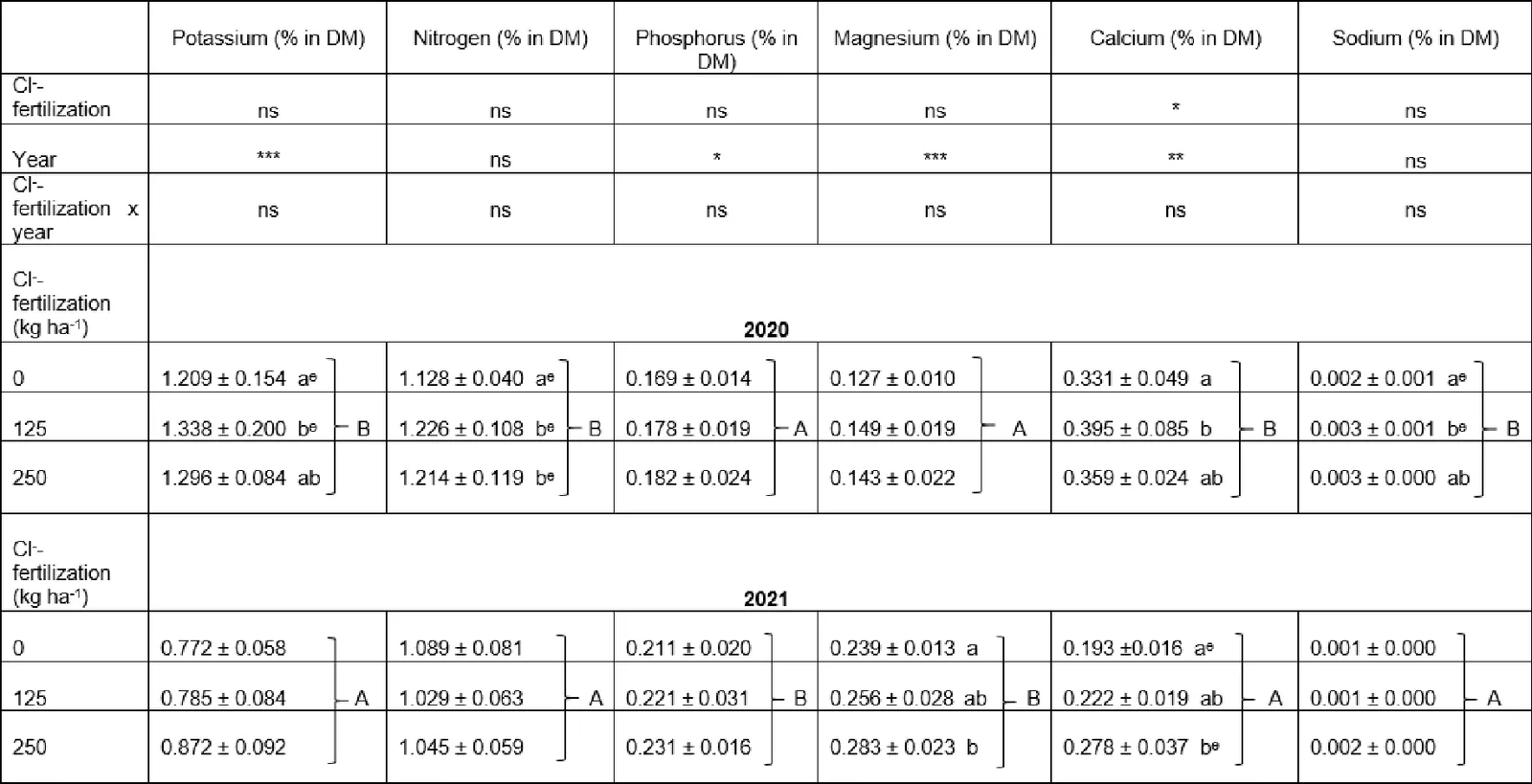
Mineral nutrient concentrations of silage maize in 2020 and 2021 under different chloride fertilization treatments. Potassium, nitrogen, phosphorus, magnesium, calcium and sodium concentrations in % in dry matter (DM) of silage maize in the years 2020 and 2021, respectively, under three different Cl^−^-fertilizations (0, 125, and 250 kg ha^−1^) (*n* = 6). Mean ± SE values. Results of the variance analyses with the fixed effects ʽ Cl^−^-fertilizationʼ, ʽyearʼ and their interactions where different stars denote p-values as follows: *p* ≤ 0.05: *, *p* ≤ 0.01: **, *p* ≤ 0.001: ***; ns denotes not significant. The results of the variance analyses are shown in the 3rd to 5th column. Small letters = significant mean differences between different Cl^−^-fertilizations where a compared to b indicates significant lower values. Capitals = significant mean differences between the years 2020 and 2021 where A compared to B indicates significant lower values. Pairwise comparisons were based on Tukey-adjusted contrasts. Letters without θ indicate differences at *p* < 0.05; a superscript θ indicates a marginal difference at 0.05 ≤ *p* < 0.10. Means sharing the same letter are not significantly different at the respective significance level. The absence of a letter indicates that there are no significant differences in means.

## Discussion

For optimal growth, most crops require Cl^−^ in the range of 0.2 to 2.0 g kg^− 1^ DM (Fig. 2)^15^. However, there are several reports that Cl^−^ can accumulate in plant tissues to a content comparable to those of macronutrients without causing salt injuries, reaching 20 g kg^− 1^ DM^11,17^, or even 50 g kg^− 1^ DM in tobacco^12^(*Nicotiana tabacum* L. var. Habana). For maize (*Zea mays* L.), Zhang et al.^18^ reported a Cl^−^-content up to 8.5 g kg^− 1^ DM in leaves without showing any salt stress symptoms. This implies that 4.6 g Cl^−^ kg^− 1^ DM silage maize, which was the results of the 250 kg Cl^−^ ha^− 1^-fertilization in 2021 (Fig. 2a), was not harmful and did not induce Cl^−^-related damage. In accordance, there were no visual salt injuires or other growth limiting observations made as a result of the Cl^−^-fertilization in this study (data not shown). Since Cl^−^ can serve as an osmoticum^19,20^, its high accumulation in plant tissues may indicate a physiological role in maintaining cellular water balance and promoting shoot hydration. No visual salt injury or growth-limiting symptoms were observed in response to Cl^−^-fertilization within the tested range of 0 to 250 kg Cl^−^ ha^−1^.

In tobacco (*Nicotiana tabacum* L. var. Habana), the accumulation of Cl^−^ in leaves has been shown to lower the osmotic potential, leading to increased leaf water contents, increased leaf cell-expansion and finally higher leaf biomasses^10,19^. Our results indicate that Cl^−^-fertilization enhanced plant water content in 2021, but not in 2020, despite comparable increases in tissue Cl^−^-content across both years, but with higher absolute values in 2021 (Fig. 2a). This suggests that the beneficial effects of Cl^−^ on plant water status depend not only on the amount of Cl^−^ applied but also on environmental conditions that faciliate the uptake of Cl^−^. Chloride is taken up mainly via massflow^15^, thus, a sufficient soil water status and proper transpiration is prerequisite. Higher precipitation in spring 2021, especially in March (Fig. 1), likely eased more effective Cl^−^-uptake via mass flow, enabling its osmotic function in maintaining tissue hydration. In contrast, the drier conditions in 2020 from March to May (Fig. 1) may have limited its impact on leaf hydration, despite measurable Cl^−^-accumulation in leaf tissue: Due to the change in soil water under dry conditions, water uptake was likely to be impeded in the plant. This interaction highlights how important sufficient water availability early in the season is for Cl^−^ to work effectively as an osmoticum during later drought stress in Brandenburg. It is assumed that at least Cl^−^-contents higher than 3.2 g kg^− 1^ DM (treatment 125 kg Cl^−^ ha^− 1^ in 2021, Fig. 2a) result in higher plant water contents, provided there is sufficient soil water.

Higher shoot hydration can be of advantage during drought but also during heat stress periods. In Fig. 1, it can be seen that the months June and July in 2021 were not only marked by drought but also by heat. The month June was 3 °C hotter than the long-term average 1991–2020 for this month with maximum temperatures up to 35 °C and the month July 1.3 °C hotter with maximum tempeatures up to 30 °C. Lobell et al.^21^ investigated how previous climatic changes in the period 1980–2008 have affected maize yields in major growing regions. Although maize is a C4 plant, Lobell et al.^21^ demonstrated that maize suffered a 3.8% yield decline in the years 1980–2008 in comparison to a world without global warming. With respect to this, it is likely, that a Cl^−^-accumulation in leaves contributes to the resilence of maize to higher temperatures: Due to increased plant water contents following the Cl^−^-accumulation, provided that the spring provided sufficient soil water reserves, plants may have a larger reservoir of water which can be used for cooling and maintaining metabolism.

A more efficient use of water based on the osmotic properties of Cl^−^ is also refelected in the rain use efficiency (RUE, calculated with the fresh mass harvest yield) (Fig. 3b): In 2021, the RUE is significant higher (*p* < 0.1) in plants treated with 125 and 250 kg Cl^−^ ha^− 1^, respectively, compared to control plants. The observations of 2021 suggest that Cl^−^-fertilization improved the efficiency of water use under less favorable conditions. The RUE shows overall significant higher values for the year 2020 due to the overall higher yield level in this year. Similar to our findings, Franco-Navarro et al.^12^ found an increased integrated water use efficiency assessed as the increase in plant fresh weight over time relative to cumulative water consumption water cocsumption (g FW ml H_2_O^–1^). However, the presented study extends these findings by demonstrating under real field conditions and suboptimal water supply that Cl^−^-fertilization can enhance water use efficiency and yield performance—highlighting its practical potential for drought-prone regions.

In 2020, there was no significant effect of the fertilization treatment on the fresh mass harvest yield (dt FM ha^− 1^) (Fig. 3a). Besides, the year 2020 had an overall and significant higher yield level compared to 2021 (Fig. 3a). This may be attributed to the relatively high precipitation in June 2020, which averaged 64.7 mm, i.e. more than 10 mm above the long-term average for June from 1991 to 2020 (53.8 mm) (Fig. 1). In addition to a wet June, most of rainfall in July happened in the first half of the month (28.5 mm; Supplementary File 1), while only 5.3 mm fell during the second half of the month. As a result, in 2020, sufficient precipitation likely occurred before the onset of flowering on July 19; which is a developmental stage that is highly sensitive to drought in maize^6^, enabling the soil to retain enough water to compensate for the comparatively dry period. (Fig. 1).

Maize plants supplied with 250 kg Cl^−^ ha^− 1^ achieved identical fresh mass yields in both years (411 dt FM ha^− 1^ in 2021 and 412 dt FM ha^− 1^ in 2020). Of note, however, the improved water status of maize plants in 2021 could not be translated into an increase in dry matter substance: The Cl^−^-fertilization did not have an significant impact on the dry mass yield in both years (Fig. 3c). This is maybe because increased water content mainly enhanced tissue hydration without stimulating carbon assimilation or biomass accumulation. Additionally, low soil levels of especially N and K across treatments (Table 1) may have limited dry matter formation despite improved water status. According to Bergmann^22^ sufficient plant content of Nand K should be at least 2.8%and 2.0%, respectively. Thus, especially in 2021, the plants showed N and K values up to 60–65% lower than considered sufficient (Table 2). This means the higher fresh mass yield of plants treated with 250 kg Cl^−^ ha^− 1^ in 2021 can be attributed simply to the higher water contents of these plants. Still, in case of the present study, it can be stated that the increased shoot water content may have improved the quality of silage maize with respect to dry matter content (%) (Supplementary File 2) for the following reason: Optimal dry matter contents should be in the range of 30–35%^23^. Several studies demonstrated that drought stress can result in increased dry matter contents of silage maize (for instance Zi et al.^24^ and Yan et al.^25^, what may result in problems with compaction and fermentation during the silage process. As can be seen in Supplmentary File 2, the dry matter content in the year 2020 was with 38.1% (250 kg Cl^−^ ha^− 1^) to 40.2% (control plants) dry matter clearly above the optimum of 30–35% dry matter. Similarly, in 2021 control plants revealed dry matter contents above 36%. These relative high dry matter contents are likely the result of drought stress in the years 2020 and 2021. However, in 2021, plants treated with 125–250 kg Cl^−^ ha^− 1^, respectively, exhibited dry matter contents of around 31%.

Chloride accumulated to near-macronutrient levels when comparing with that of Ca. Under the 125 and 250 kg Cl^−^ ha^− 1^ treatments, Cl^−^ equals or even exceeds the Ca content (Table 1). Thus, it is not surprising that this influenced the balance of other mineral nutrients. The increasing Ca content with increasing Cl^―^-fertilization is the result of the fact that Cl^−^ was fertilized via calciumchloride (CaCl_2_). As a negatively charged ion, Cl^−^ uptake requires cationic counterbalance to maintain cellular electroneutrality. Accordingly, with increasing Cl^−^-fertilization, there was a trend toward higher cation content, with significant effects for Ca in both years, Mg in 2021, and K in 2020 (Table 1). While K and Ca likely acted as primary counterions in 2020, Mg appeared to take this role in 2021 (Table 1). It can be speculated that the increase in Mg content in 2021 is attributable to the wetter spring that depleted exchangeable K in the sandy soil and simultaneously increased Mg mobility, making Mg the key counterion for the extra Cl^−^ absorbed. Clay carries a negative charge that attaches cations in two distinct layers around the clay: a thin inner layer, and a more loosen outer layer called the “diffuse double layer”. When rainfall dilutes the ion strength of the soil solution, also the content of the cations in the outer layer falls. With fewer ions available to mask the surface charge, the outer ionic cloud attrackts more cations that are further away from the particle. Since cations like Mg is no adsorbed to the clay at a greater distance, the binding force holding them weakens, making it easier for ions like Mg to detach from the clay surface and enter the soil solution, from where it becomes available to plants via mass flow^26^.

Content of the macronutrients N, K and P were below the optimum values reported by Bergmann^22^ even in the control, a pattern that reflects the nutrient-poor sandy soils typical for Brandenburg, Germany. The moderate rise in shoot water status under 250 kg Cl^−^ ha^− 1^ (Fig. 2b) may have supported the slightly higher cation uptake shown in Table 2 as a better plant water status is likely the effect of increased water uptake via mass-flow, being a driving factor for uptake of nutrients such as N or K. Yet, this positive effect was not strong enough to compensate for the overall lack of essential macronutrients.

## Conclusion

Our experiment showed that a Cl^−^-fertilization (250 kg Cl^−^ ha^−1^) raised shoot Cl^−^-levels in maize under field conditions and increased fresh biomass yields in the year 2021 that was characterized by sufficient rainfall during spring. We suggest that this effect may have resulted from Cl^−^-induced improvements in plant water relations, possibly supported by sufficient groundwater availability, and was reflected in higher shoot water content and enhanced rain-use efficiency at elevated shoot Cl^−^ levels. Thus, the present study supports on the role of Cl^−^ as an osmoticum in plants, demonstrating its function in osmotic adjustment at concentrations within the macronutrient range. The drier spring of 2020 likely limited these effects because soil water availability was insufficient, even though plants still accumulated Cl^−^. We suggest that—for Cl^−^ to support plant water status and yield under summer drought conditions – enough moisture must be available earlier in the season to allow sufficient Cl^−^ uptake.

## Supplementary Information

Below is the link to the electronic supplementary material.

Supplementary Material 1

Supplementary Material 2

## Data Availability

Data will be provided upon request. To obtain data please contact MR.
