# Supplementary material for: Chloride fertilization increased maize shoot chloride levels and yield depending on soil moisture conditions
Source: Sci Rep. 2026 Aug 25;16:26680. doi: 10.1038/s41598-026-68369-x (PMC13507284; doi:10.1038/s41598-026-68369-x)
Supplement: Supplementary file 1 [file 41598_2026_68369_MOESM2_ESM.docx]

**Supplementary File 2:** Dry mass content in % at harvest in the years 2020 and 2021 under three different chloride fertilizations (0, 125, and 250 kg ha^-1^). n = 6, mean ± SE values. Results of the variance analyses with the fixed effects ʽ Cl^―^-fertilizationʼ, ʽyearʼ and their interactions; significance levels are indicated by asterisks as follows:: p ≤ 0.05: *, p ≤ 0.01: **, p ≤ 0.001: ***; ns denotes not significant. Small letters above the bars = significant mean differences between different chloride fertilizations where ʽaʼ signifies significant lower values compared to ʽbʼ. Capitals= significant mean differences between the years 2020 and 2021 where ʽAʼ signifies significant lower values compared to ʽBʼ. Level of significance for mean differences determined by multiple contrast tests is p < 0.05 or p < 0.1, as indicated to a supersripted ᶱ attached to capital letters. The absence of a letter indicates that there are no significant differences in means.
